# Different Spatial Configurations of LED Light Sources Enhance Growth in Tomato Seedlings by Influencing Photosynthesis, CO_2_ Assimilation, and Endogenous Hormones

**DOI:** 10.3390/plants14091369

**Published:** 2025-04-30

**Authors:** Xiting Yang, Shuya Wang, Wenkai Liu, Shuchao Huang, Yandong Xie, Xin Meng, Zhaozhuang Li, Ning Jin, Li Jin, Jian Lyu, Jihua Yu

**Affiliations:** 1 College of Horticulture, Gansu Agricultural University, Lanzhou 730070, China; yangxiting22yjs@163.com (X.Y.); 18093668927@163.com (W.L.); 18894335763@163.com (S.H.); xieyandongsx@163.com (Y.X.); mx17393157902@163.com (X.M.); 18298405838@163.com (Z.L.); jinn0513@163.com (N.J.); 2 State Key Laboratory of Aridland Crop Science, Gansu Agricultural University, Lanzhou 730070, China; wsyhn95@163.com (S.W.); jinli0124@163.com (L.J.)

**Keywords:** *Solanum lycopersicum* L., LED supplemental lighting, photosynthetic characteristics, spatial light arrangement, carbon metabolism

## Abstract

Sub-optimal light environments in controlled agricultural settings often limit the productivity of plants. While LED supplementary lighting has been widely adopted to mitigate light deficiencies, the spatial arrangement of LED light sources remains a critical but under-explored factor affecting plant physiological responses. In this study, we used the affiliation function method to comprehensively analyze the effects of four spatial LED supplementary lighting configurations—top-down lighting (T1), mid-canopy upward lighting (T2), mid-canopy downward lighting (T3), and bottom-up lighting (T4)—on the growth and photosynthetic performance of tomato plants. Our findings reveal that the T1 treatment significantly increased light absorption in the upper and middle leaves, enhanced photosynthetic efficiency, promoted the CO_2_ assimilation rate, and elevated the activities of key Calvin cycle enzymes, including ribulose-1,5-bisphosphate carboxylase/oxygenase (Rubisco), fructose-1,6-bisphosphatase (FBPase), transketolase (TK), glyceraldehyde-3-phosphate dehydrogenase (GAPDH), and fructose-1,6-bisphosphate aldolase (FBA). These changes led to improved carbohydrate metabolism and biomass accumulation. Additionally, the T4 treatment markedly enhanced photosynthetic activity in the lower leaves, increasing sugar metabolism-related enzyme activities, such as sucrose synthase (SS), sucrose phosphate synthase (SPS), acid invertase (AI), and neutral invertase (NI). Consequently, compared with the CK treatment, the T4 treatment significantly increased the accumulation of glucose, fructose, and sucrose, with increases of 47.36%, 27.61%, and 87.21%, respectively. Furthermore, LED supplementation regulated endogenous hormone levels, thereby promoting overall plant growth. This study highlights the importance of the spatial arrangement of LEDs in optimizing light distribution and enhancing plant productivity, providing valuable theoretical and practical insights for improving agricultural practices in controlled environments.

## 1. Introduction

Tomato (*Solanum lycopersicum* L.)—an herbaceous plant cultivated as an annual or perennial crop—is globally valued for its nutritional content, distinct flavor, and dual classification as both a fruit and vegetable. As one of the most significant vegetable crops, it serves as a primary winter crop in solar greenhouses. However, winter cultivation presents challenges such as low temperatures, short photoperiods, and frequent cloudy days, which adversely affect crop growth [1,2]. Light serves not only as the primary energy source for photosynthesis, but also as a developmental signal regulating photomorphogenesis [3]. Consequently, light is a critical environmental factor influencing the growth of crops in greenhouses, and the design of a greenhouse—including orientation, roof angle, and covering materials—significantly influences its indoor light environment. These challenges are exacerbated by aging greenhouse coverings, the accumulation of dust, and improper management of insulation quilts (e.g., delayed uncovering and premature covering), collectively creating sub-optimal light conditions. Such conditions result in excessive seedling elongation, leaf chlorosis, severe flower and fruit abscission, delayed fruit maturation, and elevated pest and disease incidence, ultimately leading to substantial yield losses [4]. Furthermore, a high planting density further exacerbates light insufficiency conditions [5].

Supplemental lighting is a widely adopted strategy to mitigate light limitations in solar greenhouse vegetable cultivation, which has emerged as a pivotal research focus in recent years. Light-emitting diodes (LEDs) have been recognized as an optimal light source for greenhouse applications due to their capacity for precise regulation of light intensity, spectrum, and photoperiod tailored to plant requirements [6,7]. As a cool light source, LEDs offer distinct advantages such as energy efficiency, environmental sustainability, compactness, durability, and customizable spectral outputs [8]. Their close-range irradiation capability further optimizes spatial utilization. Under LED lighting, plants can achieve typical growth and developmental processes, establishing LEDs as a practical solution to counteract insufficient winter greenhouse illumination [9]. Additionally, LED systems facilitate intensive crop management and standardized cultivation practices, advancing modern agricultural efficiency. Within the visible spectrum (400–700 nm), blue (B) and red (R) light wavelengths exhibit the highest chlorophyll absorption rates and are the most physiologically active for plant growth [10]. These spectra provide energy for photosynthetic carbon dioxide (CO_2_) assimilation while regulating growth, cellular differentiation, and the synthesis of secondary metabolites [11]. Mixed red–blue (RB) light has been extensively utilized as a standard spectrum in (semi-)closed plant production systems [12]. Research has demonstrated that optimal RB ratios significantly enhance plant growth, primarily through increased chlorophyll a (Chl *a*), chlorophyll b (Chl *b*), total chlorophyll (Total Chl), and electron transport rate (ETR), alongside the rapid induction of non-photochemical quenching (NPQ) [13]. These adaptations improve the photosynthetic efficiency and activity of plants through the modulation of carbohydrate metabolism [14,15].

Recent advancements in LED supplemental lighting technology have enabled the development of diverse spatial configurations to optimize greenhouse crop growth, including top lighting, where LEDs suspended above the canopy significantly enhance photosynthetic rates in the upper leaves, improving plant architecture and overall growth [16,17]; however, limited light penetration of the middle and lower foliage reduces overall photosynthetic efficiency. In inter-canopy lighting, LEDs are positioned between plant rows or within the canopy, thus improving photosynthetic efficiency and light utilization in the middle and lower leaves. This configuration enhances chlorophyll content, fruit quality, and fruit set while creating uniform light distribution, increasing yields by up to 50% [18,19]. Compared to high-pressure sodium lamps, inter-canopy LED lighting reduces energy consumption by 36.3% [20,21]. For bottom lighting, ground-installed LEDs are used to improve the light environment for lower leaves, significantly boosting stomatal conductance and net photosynthetic rate while delaying senescence [22]. This method accelerates fruit ripening and photosynthetic product accumulation, achieving the highest energy efficiency under the same power conditions [23].

Different supplemental lighting methods (i.e., top, inter-canopy, and bottom lighting) influence the optimization of the light environment and crop growth. To investigate these effects, *Solanum lycopersicum* L. ‘Jingfan 502’ was used as the experimental cultivar in this study. A pot experiment was conducted in a modern greenhouse using red–blue LED growth lights (7R2B) as the supplemental source. Lighting was applied to the inter-canopy from 6:00 to 9:00 and 17:00 to 20:00, with four spatial configurations: T1 (top lighting above the canopy), T2 (inter-canopy upward lighting), T3 (inter-canopy downward lighting), and T4 (bottom lighting). Photosynthetic characteristics, carbohydrate accumulation, Calvin cycle enzyme activities, and sugar metabolism-related enzyme indicators were analyzed to evaluate the differential photosynthetic responses of tomatoes to the LED supplemental lighting arrangements. This research provides theoretical insights for the application of LED supplemental lighting in greenhouse vegetable production.

## 2. Results

### 2.1. Accumulation of Biomass Under Various Spatial Arrangements of LED Light Sources

**Figure 1:**
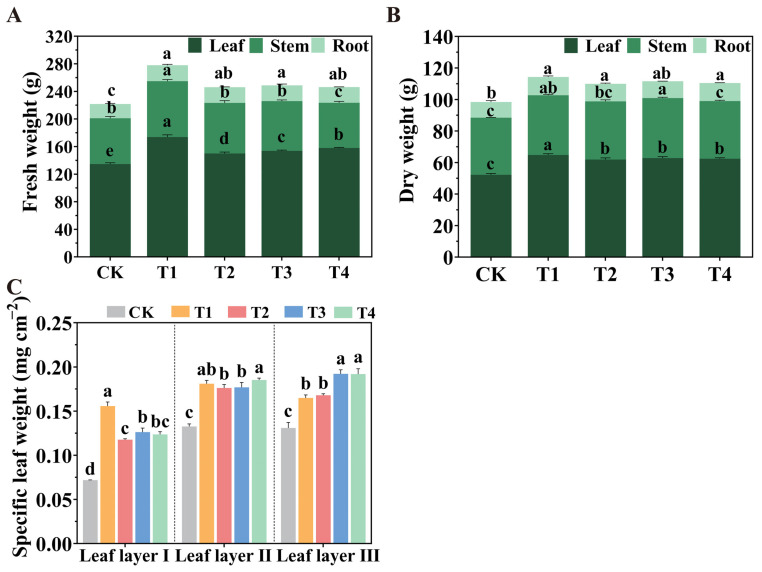
Effects of different spatial arrangement of LED supplemental lights on the (**A**) fresh weight, (**B**) dry weight and (**C**) SLW of tomato seedlings after 30 days of treatment. Values are presented as the mean ± SD (n = 3), with three independent biological replicates. For each biological replicate, at least six leaves were harvested. Statistical differences between treatments, determined at a significance level of *p* < 0.05, are indicated by different lowercase letters. Standard errors are shown as error bars.

The effects of different spatial configurations of LED light sources on tomato dry matter accumulation and specific leaf weight (SLW) are presented in Figure 1. Compared to the CK treatment, all LED treatments significantly increased the fresh weights of leaves and roots (Figure 1A). Among the treatments, the T1 treatment notably enhanced stem fresh weight. Following the T1 treatment, the dry weights of leaves, stems, and roots were significantly higher, while the T3 treatment significantly increased the dry weights of leaves and stems (Figure 1B). All spatial configurations of LED supplemental lighting significantly improved the SLW of tomato leaves across all layers (Figure 1C). Specifically, the T1 treatment significantly increased the SLW of leaf layer I by 116.89% compared to the CK treatment; meanwhile, the T4 treatment substantially enhanced the SLW of leaf layer II, with increases of 39.87%, 4.93%, and 4.61% compared to the CK, T2, and T3 treatments, respectively. For leaf layer III, the T3 treatment significantly increased SLW by 46.92%, 16.64%, and 14.44%, while the T4 treatment resulted in increases of 46.54%, 16.34%, and 14.14% compared to the CK, T1, and T2 treatments, respectively. Additionally, the T1 and T2 treatments significantly improved SLW by 25.96% and 28.39% compared to CK treatment, respectively, with no significant difference between T1 and T2 treatments.

### 2.2. Spatial Arrangement of LED Light Sources Promotes Photosynthetic Pigment Accumulation

**Figure 2:**
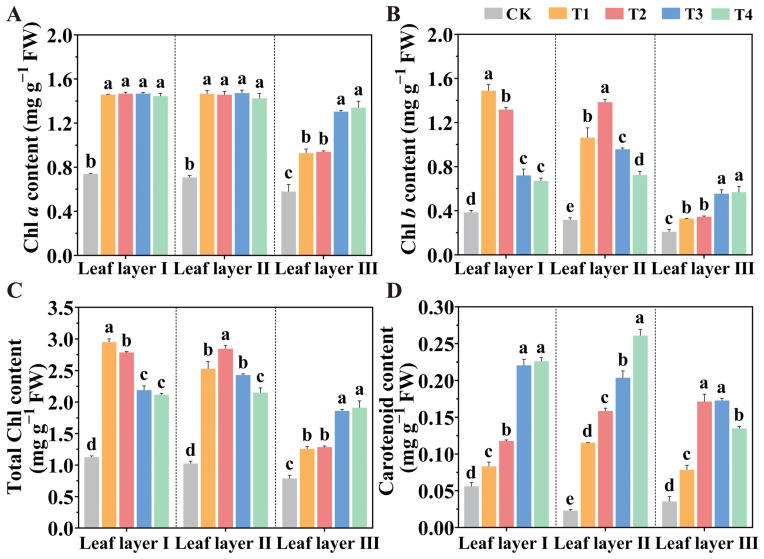
The spatial arrangement of LED supplemental lights influenced the photosynthetic pigment content in tomato plants. This included (**A**) Chl *a*, (**B**) Chl *b*, (**C**) Total Chl, and (**D**) Car contents in tomato leaves after light treatments for 30 d. Values are presented as the mean ± SD (n = 3), with three independent biological replicates. For each biological replicate, at least six leaves were harvested. Statistical differences between treatments, determined at a significance level of *p* < 0.05, are indicated by different lowercase letters. Standard errors are shown as error bars.

Photosynthetic pigments are crucial for plants to absorb light and perform photosynthesis. To explore the impacts of different spatial arrangements of LED light sources on these pigments, we assessed the pigment contents in tomato leaves. The results demonstrated that all supplemental light treatments significantly increased the contents of Chl *a* (Figure 2A), Chl *b* (Figure 2B), Total Chl (Figure 2C), and carotenoids (Car) (Figure 2D) across different leaf layers, when compared to the CK treatment. Specifically, treatments T1 and T2 significantly enhanced Chl *b* content in leaf layer I by 106.58% and 82.67%, respectively, when compared to T3 treatment. In leaf layer II, the T1 and T2 treatments also significantly increased Chl *b* content by 11.04% and 44.67%, respectively. Additionally, both the T3 and T4 treatments significantly increased Chl *a*, Chl *b*, Total Chl, and Car contents in leaf layer III, compared to the CK, T1, and T2 treatments. These results indicated that the spatial arrangement of LED supplemental lighting can markedly enhance the contents of photosynthetic pigments in tomato leaves, thereby boosting the leaves’ ability to absorb light and transfer energy efficiently.

**Figure 3:**
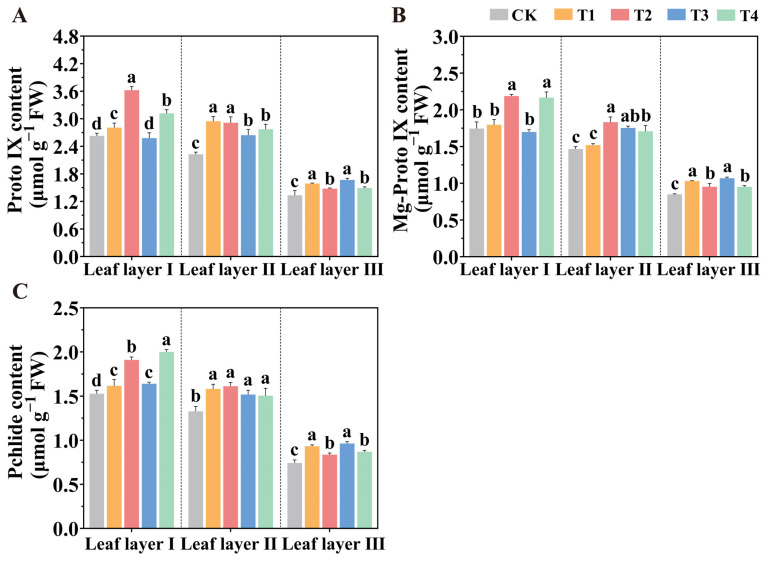
The contents of Proto IX (**A**), Mg-Proto IX (**B**) and Pchlide (**C**) in tomato seedlings after light treatments for 30 d. Values are presented as the mean ± SD (n = 3), with three independent biological replicates. For each biological replicate, at least six leaves were harvested. Statistical differences between treatments, determined at a significance level of *p* < 0.05, are indicated by different lowercase letters.

To investigate the reasons for the significant differences in chlorophyll content under various spatial arrangements of light sources, we analyzed the chlorophyll synthesis precursors in tomato leaves under different treatments. In leaf layer I, the T2 treatment significantly increased the Protoporphyrin IX (Proto IX) content, compared to the CK, T1, T3, and T4 treatments, with increases of 37.83%, 29.07%, 40.39%, and 16.10%, respectively (Figure 3A). Similarly, in leaf layer III, the T1 and T3 treatments also significantly elevated Proto IX content. Compared to the CK treatment, all supplemental light treatments significantly enhanced Mg-protoporphyrin IX (Mg-Proto IX) content in leaf layer III (Figure 3B). Notably, the T2 treatment increased the Mg-Proto IX content in leaf layers I and II by 25.43% and 24.92%, respectively, while the T3 treatment significantly increased this compound in leaf layer III by 25.78%. Additionally, the T2 treatment significantly enhanced Protochlorophyllide (Pchlide) content in leaf layer I, compared to the CK, T1, and T3 treatments, with increments of 24.99%, 18.09%, and 16.42%, respectively. Similarly, the T4 treatment also markedly increased Pchlide content in leaf layer I, compared to the CK, T1, and T3 treatments, with increases of 30.86%, 23.64%, and 21.89%, respectively (Figure 3C). In leaf layer III, the T1 and T3 treatments significantly elevated Pchlide content relative to CK treatment, with increases of 25.57% and 29.82%, respectively.

### 2.3. Manipulation of the Spatial Arrangement of LED Supplemental Lights Improves Photosynthesis

**Figure 4:**
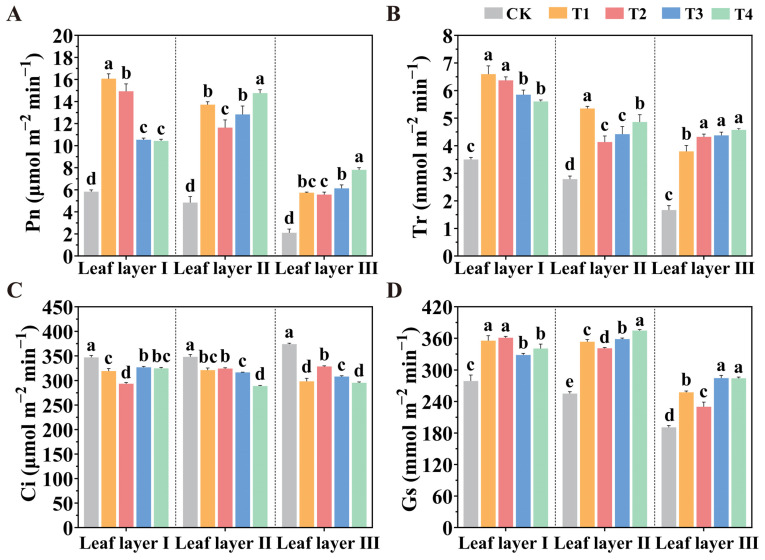
Effect of LED supplemental light source spatial arrangements on the photosynthetic characteristics of tomato leaves. Panels represent (**A**) Pn, (**B**) Tr, (**C**) Ci, and (**D**) Gs in tomato leaves after 30 days of light treatment. Values are presented as mean ± SD (n = 3), derived from three independent biological replicates. For each biological replicate, at least six leaves were sampled. Statistical differences among treatments were evaluated using a significance threshold of *p* < 0.05 and are denoted by different lowercase letters. Error bars represent standard deviations.

Gas exchange parameters provide a direct reflection of a plant’s photosynthetic capacity. Figure 4 illustrates the responses of the tomato leaf gas exchange parameters to supplemental lighting treatments with different spatial arrangements of LED light sources. Overall, compared to the CK treatment, the T1, T2, T3, and T4 treatments all significantly increased the net photosynthetic rate (Pn) and transpiration rate (Tr) across the three leaf layers of tomato seedlings, while showing a similar trend in terms of stomatal conductance (Gs). In contrast, intercellular CO_2_ concentration (Ci) exhibited an opposite trend. These results suggest that supplemental lighting with spatially arranged LEDs enhances the photosynthetic capacity of tomato leaves. In leaf layer I, Pn under the T1 treatment increased by 52.53% and 53.99%, while Tr increased by 12.77% and 17.67% when compared to the T3 and T4 treatments, respectively. Under the T2 treatment, Gs increased by 29.46% compared to the CK treatment, while Ci significantly decreased by 15.63%. In leaf layer II, the T4 treatment significantly increased Pn, Tr, and Gs by 205.52%, 74.40%, and 46.84%, respectively, compared to the CK treatment. Similarly, in leaf layer III, the T4 treatment increased Pn, Tr, and Gs by 271.59%, 174.40%, and 48.89%, respectively. Additionally, the T4 treatment markedly reduced Ci in leaf layers II and III by 16.95% and 20.99%, respectively.

### 2.4. Manipulation of the Spatial Arrangement of LED Supplemental Light Influences Chlorophyll Fluorescence

**Figure 5:**
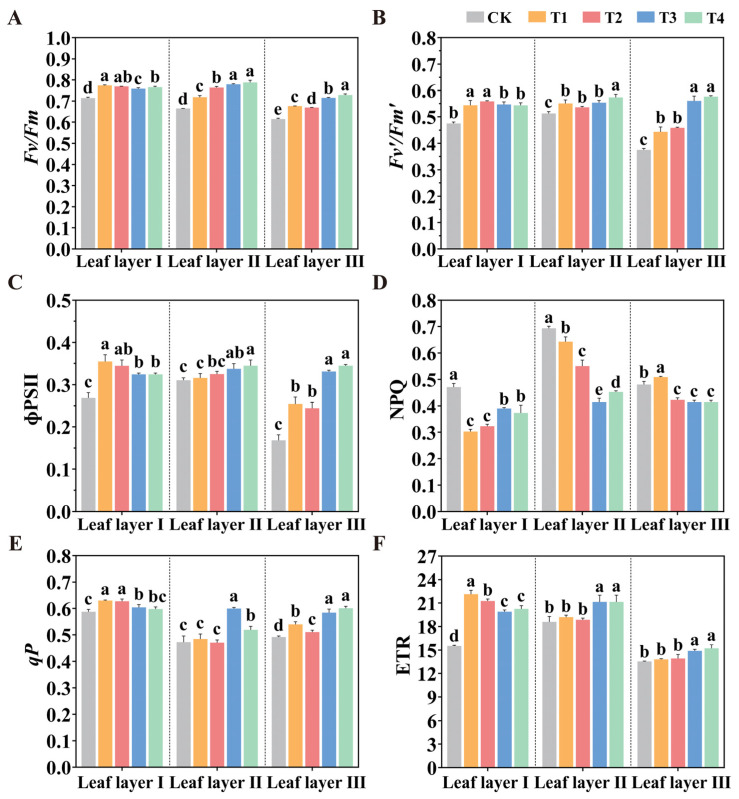
Effect of LED supplemental light source spatial arrangements on the fluorescence parameters of tomato leaves after 30 days of light treatment. (**A**) *Fv*/*Fm*; (**B**) *Fv*′/*Fm*′; (**C**) ɸPSII; (**D**) NPQ; (**E**) *qP*; (**F**) ETR. Values are presented as mean ± SD (n = 3), derived from three independent bio-logical replicates. For each biological replicate, at least six leaves were sampled. Statistical differences among treatments were evaluated using a significance threshold of *p* < 0.05 and are denoted by different lowercase letters. Error bars represent standard deviations.

Chlorophyll fluorescence parameters describe the photosynthetic mechanisms and physiological status of plants, reflecting the complex relationships between photosynthesis and the environment. Given the differences in photosynthetic pigment content observed under the different treatments, we found that the spatial arrangement of LED supplemental lights may significantly affect the photosynthetic parameters of tomato seedlings. Therefore, we analyzed the changes in fluorescence parameters to assess the impacts of different LED light spatial arrangements on chlorophyll fluorescence in tomato leaves (Figure 5). The results showed that the different LED spatial arrangements significantly enhanced several chlorophyll fluorescence parameters in tomato leaves, including the maximum photochemistry quantum yield of PSII (*Fv*/*Fm*), the efficiency of excitation energy captured by open PSII reaction centers (*Fv*′/*Fm*′), the photochemical quenching coefficient (*qP*), the effective quantum yield of PSII photochemistry (ɸPSII), and ETR. In contrast, NPQ in photosystem II was significantly reduced. In leaf layer I, the T1 treatment significantly increased *Fv*/*Fm*, *Fv*′/*Fm*′, ɸPSII, *qP*, and ETR compared to the CK treatment, with increases of 8.31%, 14.54%, 32.09%, 7.09%, and 42.49%, respectively, while significantly reducing NPQ by 35.84%. Similarly, the T3 and T4 treatments significantly enhanced ETR, *Fv*/*Fm*, *Fv*′/*Fm*′, and ɸPSII in leaf layers II and III, while significantly reducing NPQ in these layers. These data indicate that different spatial arrangements of LED light sources may increase the proportion of open PSII reaction centers during adaptation to light, thus enhancing PSII activity and improving the maximum photochemical efficiency and electron transport capacity, which is consistent with the observed increases in photosynthetic pigment contents.

### 2.5. Manipulation of the Spatial Arrangement of LED Supplemental Light Influences Calvin Cycle Enzyme Activities

**Figure 6:**
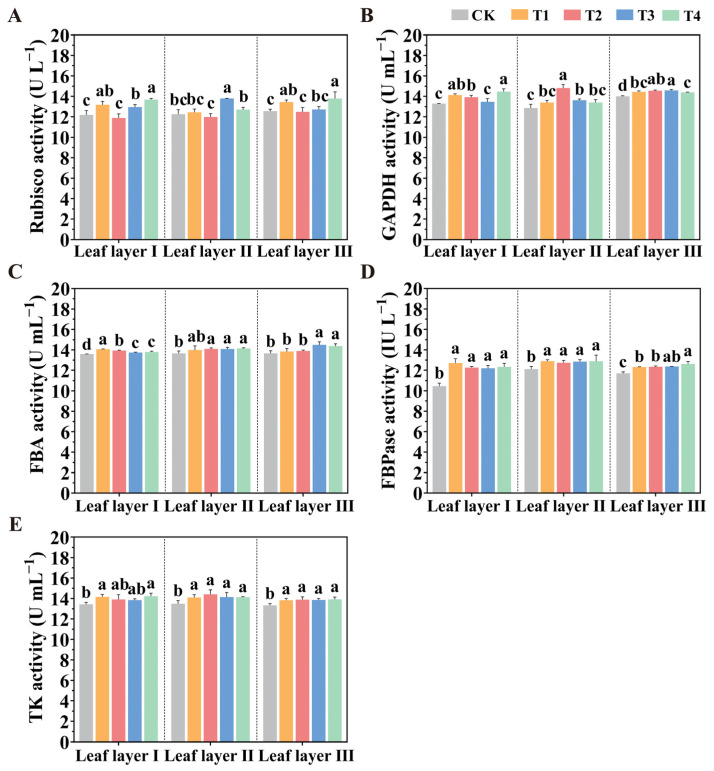
Effect of spatial arrangements of LED supplemental light sources on the activity of Calvin cycle-related enzymes in tomato leaves after 30 days of light treatment. (**A**) Rubisco; (**B**) GADPH; (**C**) FBA; (**D**) FBPase; (**E**) TK activities. Values are presented as mean ± SD (n = 3), derived from three independent biological replicates. For each biological replicate, at least six leaves were sampled. Statistical differences among treatments were evaluated using a significance threshold of *p* < 0.05 and are denoted by different lowercase letters. Error bars represent standard deviations.

The activities of key enzymes in the Calvin cycle directly influence the photosynthetic capacity of plants. As shown in Figure 6, in leaf layer I, the ribulose-1,5-bisphosphate carboxylase/oxygenase (Rubisco) activity under the T4 treatment was significantly higher by 12.20%, 15.04%, and 5.54% when compared to the CK, T2, and T3 treatments, respectively. For the glyceraldehyde-3-phosphate dehydrogenase (GAPDH) activity in the same leaf layer, the T1, T2, and T4 treatments increased activity by 5.05%, 3.45%, and 7.49% compared to the T3 treatment and by 6.23%, 4.62%, and 8.70% compared to the CK treatment. In terms of fructose-1,6-bisphosphate aldolase (FBA) activity, the T1 treatment increased activity by 2.37% and 1.96% compared to the T3 and T4 treatments, respectively, while the T3 and T4 treatments increased activity by 1.17% and 1.57% compared to the CK treatment. For the fructose-1,6-bisphosphatase (FBPase) activity in leaf layer I, all LED treatments significantly increased activity (by 21.58%, 17.24%, 16.60%, and 18.02%, respectively) compared to the CK treatment. The activity of transketolase (TK) in leaf layer I was also significantly enhanced by the T1 and T4 treatments, with increases of 3.08% and 5.98%, respectively, compared to the CK treatment. In leaf layer II, compared to the CK treatment, all LED treatments significantly increased FBPase activity (by 6.44%, 5.09%, 6.07%, and 6.35%, respectively), while TK activity was increased by 4.62%, 6.95%, 4.90%, and 4.81%, respectively. In leaf layer II, the T2 treatment significantly increased GAPDH activity by 15.13%, 10.64%, 8.84%, and 10.63%, compared to the CK, T1, T3, and T4 treatments, respectively. For Rubisco activity in leaf layer II, the T3 treatment significantly enhanced it by 12.50%, 10.78%, 15.06%, and 8.52%, compared to the CK, T1, T2, and T4 treatments, respectively. In leaf layer III, all LED treatments significantly increased the activities of GAPDH, FBPase, and TK compared to the CK treatment. These results indicate that different spatial configurations of LED supplemental lighting can enhance the activities of key enzymes in the Calvin cycle, thus promoting CO_2_ fixation and improving the photosynthetic capacity of tomatoes.

### 2.6. Carbohydrate Accumulation Under Various Spatial Arrangements of LED Supplemental Lighting

**Figure 7:**
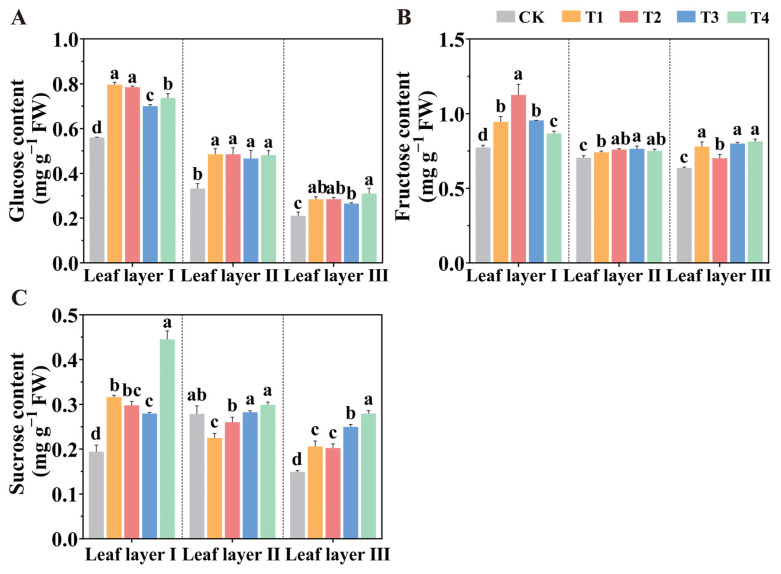
Effect of spatial arrangements of LED supplemental light sources on the contents of (**A**) glucose; (**B**) fructose and (**C**) sucrose in tomato leaves after 30 days of light treatment. Values are presented as mean ± SD (n = 3), derived from three independent biological replicates. For each biological replicate, at least six leaves were sampled. Statistical differences among treatments were evaluated using a significance threshold of *p* < 0.05 and are denoted by different lowercase letters. Error bars represent standard deviations.

Sugars, as products of photosynthesis, play a crucial role in the overall metabolic processes of plants. Figure 7 presents the accumulation of photosynthetic products in leaf layers I, II, and III of tomato plants under different LED light source spatial arrangements. The results indicate that, compared to the CK treatment, the T1 treatment significantly increased the glucose content in leaf layer I by 42.02%, while the T2 and T4 treatments significantly increased the fructose and sucrose contents in leaf layer I by 45.69% and 129.19%, respectively. The LED light source spatial arrangements significantly increased the glucose and fructose contents in leaf layer II, with glucose content increasing by 46.60%, 46.65%, 40.79%, and 45.51%, and fructose content increasing by 4.88%, 7.48%, 8.31%, and 6.24%, respectively. Compared with the CK treatment, the T4 treatment significantly increased the glucose, fructose, and sucrose contents in leaf layer III, with increases of 47.36%, 27.61%, and 87.21%, respectively.

**Figure 8:**
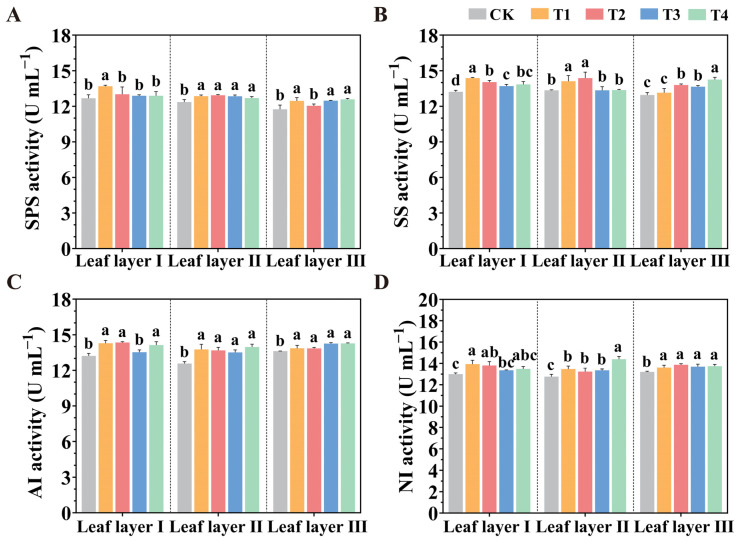
Effect of spatial arrangements of LED supplemental light sources on the activity of sugar metabolism-related enzymes in tomato leaves after 30 days of light treatment. (**A**) SPS; (**B**) SS; (**C**) AI; (**D**) NI activities. Values are presented as mean ± SD (n = 3), derived from three independent biological replicates. For each biological replicate, at least six leaves were sampled. Statistical differences among treatments were evaluated using a significance threshold of *p* < 0.05 and are denoted by different lowercase letters. Error bars represent standard deviations.

As shown in Figure 8, in leaf layer I, compared to the CK treatment, the T1 treatment significantly increased the activities of sucrose phosphate synthase (SPS), sucrose synthase (SS), acid invertase (AI), and neutral invertase (NI) by 7.94%, 8.75%, 8.10%, and 7.16%, respectively. In leaf layer II, compared to the CK treatment, the T2 treatment increased the activities of SPS, SS, and AI by 4.68%, 7.65%, and 8.84%, respectively, with no significant difference between the T1 and T2 treatments. Meanwhile, the T4 treatment significantly increased NI activity by 12.75%. In leaf layer III, compared to the CK treatment, the different LED light source spatial arrangements increased NI activity by 3.02%, 4.98%, 3.60%, and 3.97%, respectively. 

### 2.7. Endogenous Hormone Contents in Tomato Leaves Under Various LED Light Source Spatial Configurations

**Figure 9:**
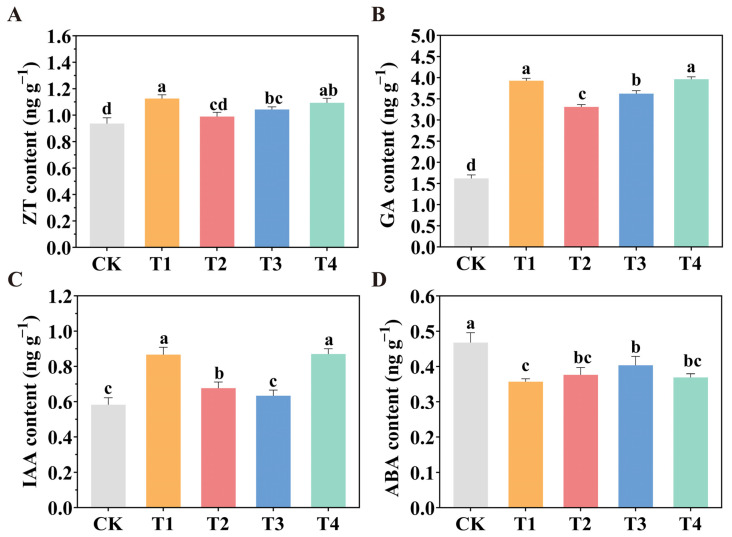
Effect of spatial arrangements of LED supplemental light sources on the plant hormones in tomato leaves after 30 days of light treatment. (**A**) ZT; (**B**) GA; (**C**) IAA; (**D**) ABA contents. Values are presented as mean ± SD (n = 3), derived from three independent biological replicates. For each biological replicate, at least six leaves were sampled. Statistical differences among treatments were evaluated using a significance threshold of *p* < 0.05 and are denoted by different lowercase letters. Error bars represent standard deviations.

As shown in Figure 9A, the zeatin (ZT) content in tomato leaves was significantly higher under the T1, T3, and T4 treatments, compared to the CK treatment, with increases of 21.48%, 12.52%, and 17.99%, respectively. As for gibberellin (GA) content, the T1 treatment significantly increased GA levels in tomato leaves by 18.70% and 8.49% compared to the T2 and T3 treatments, respectively. Similarly, the T4 treatment also significantly enhanced GA content by 19.75% and 9.44%, compared to the T2 and T3 treatments, respectively. Additionally, the T3 treatment exhibited a significant increase in GA content of 9.42% compared to the T2 treatment, while the T2 treatment showed a significant increase of 104.43% compared to the CK treatment (Figure 9B). In terms of auxin (IAA) content, the T1 treatment increased IAA levels by 27.35% and 34.56%, compared to the T2 and T3 treatments, respectively. The T4 treatment also significantly enhanced IAA levels in tomato leaves (Figure 9C). In contrast, all LED light source spatial configurations significantly reduced the abscisic acid (ABA) content in tomato leaves compared to the CK treatment, with reductions of 23.73%, 19.53%, 13.76%, and 21.10%, respectively (Figure 9D).

### 2.8. Principal Component Analyses and Affiliation Function Evaluation Analysis of Photosynthesis-Related Physiological Indices

**Figure 10:**
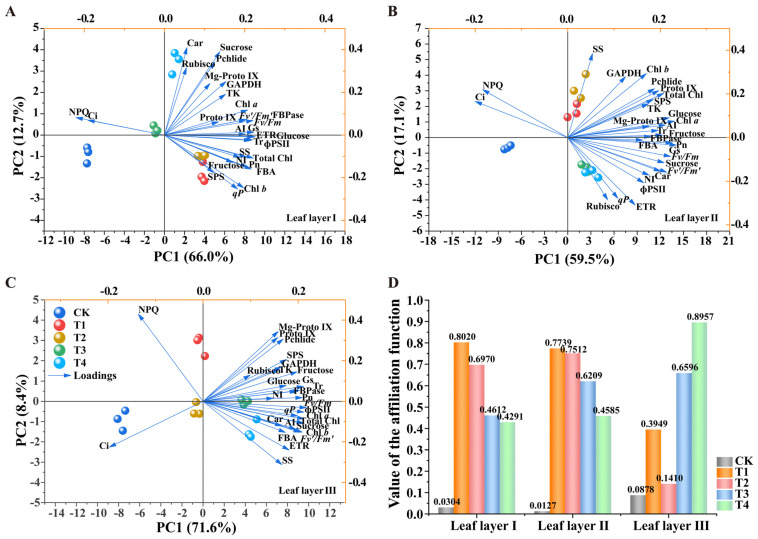
PCA of the physiological indicators in tomato for different light treatment. (**A**) Leaf layer I; (**B**) leaf layer II; (**C**) leaf layer III and (**D**) the comprehensive evaluation D value of different treatment. Values are presented as mean ± SD (n = 3), derived from three independent biological replicates. For each biological replicate, at least six leaves were sampled. Statistical differences among treatments were evaluated using a significance threshold of *p* < 0.05. The physiological indicators includ: Chl *a*; Chl *b*; Total Chl; Car; Proto IX; Mg-Proto IX; Pchlide; Pn; Tr; Ci; Gs; *Fv*/*Fm*; *Fv*′/*Fm*′; ɸPSII; NPQ; *qP*; ETR; Rubisco; GAPDH; FBA; FBPase; TK; Glucose; Fructose; Sucrose; SPS; SS; AI; NI.

The PCA method is a dimensionality reduction tool that preserves as much of the original data variation as possible [24]. PCA facilitates the exploration of differences and similarities among samples, providing an intuitive basis for comparison of samples [25]. To evaluate the effects of different settings of LED light sources on the photosynthetic capacity of tomato plants, this study classified three leaf layers using PCA. The model is illustrated in Figure 10, incorporating five light treatments and 29 photosynthesis-related physiological indicators. The PCA results revealed significant differences in physiological indicators across the three leaf layers under different supplemental light treatments. In leaf layer I, PCA explained 78.7% of the total variance, with PC1 and PC2 accounting for 66.0% and 12.7%, respectively. The primary contributors to PC1 were ETR, glucose, and ɸPSII, while carotenoids, Rubisco activity, and sucrose contributed most to PC2 (Figure 10A). The sample groups were clearly divided into two categories in PC1. One group included T1, T2, and T4 treatments, and the second category included the CK and T3 treatments. In leaf layer II, the components of PC1 and PC2 explained 76.6% of the total variation (PC1: 59.5%; PC2: 17.1%), indicating that the model correctly predicted the data. FBPase activity, Pn, fructose, Gs, and FBA activity were the main contributors to PC1, while SS activity dominated PC2 (Figure 10B). In leaf layer III, PCA explained 80.0% of the total variance, with PC1 and PC2 contributing 71.6% and 8.4%, respectively. It showed that the two main components account for the majority of all quality indicator information. Pn, NI activity, *Fv/Fm*, and FBPase activity were the primary contributors to PC1, while NPQ was the primary contributor to PC2 (Figure 10C). These results indicate that PC1 tended to capture most of the photosynthesis-related information, with significant separation among CK, T1, T2, T3, and T4 treatments in PC1, forming distinct clusters. Furthermore, the acute angles among the 27 indicators except NPQ and Ci indicated positive correlations across in different layers.

The PCA classifications were consistent with the cluster analysis results, which grouped the five treatments into three main categories: the T3 and T4 treatments formed one category, the T1 and T2 treatments another, and the CK treatment formed the third (Figure S1). In leaf layer I, the T2 treatment significantly reduced Ci content and Rubisco activity (Figure S1A). In leaf layer II, the T4 treatment reduced Ci content compared to the CK treatment, while the T3 treatment increased *qP* and Rubisco activity, and the T2 treatment enhanced GAPDH activity (Figure S1B). In leaf layer III, the T1 treatment increased ɸPSII content compared to the CK treatment, while the T4 treatment led to higher Rubisco activity than the T1, T2, and T3 treatments (Figure S1C). These findings demonstrate the differential effects of supplemental light treatments on the photosynthetic indicators of tomato leaves across different leaf layers. A comprehensive evaluation was performed using PCA and membership function analysis. PCA of 29 indicators across the three leaf layers under the five treatments revealed that the eigenvalues of the first three principal components were greater than 1, with cumulative variance contribution rates of 94.694%, 95.621%, and 95.938% for leaf layers I, II and III, respectively. This indicates that the extracted principal components effectively represent the overall variability of the data. Based on the comprehensive scores (Xi) derived from PCA, membership function values (μi) were calculated, and D values for comprehensive evaluation were obtained (Table S1). Figure 10D displays the results of the combined correlation and comprehensive evaluation analyses. Based on the D values, the treatments were ranked as follows: for leaf layers I and II, the order was T1 > T2 > T3 > T4 > CK; for leaf layer III, the order was T4 > T3 > T1 > T2 > CK. These rankings highlight the differential impacts of various light treatments on photosynthetic performance across the three leaf layers.

## 3. Discussion

Light serves as the primary energy source for plant growth. Processes such as plant development, flowering, and fruiting are regulated by photosynthesis, photomorphogenesis, and photoperiodic responses, all of which are light-dependent [26,27]. Key light-related parameters include spectral quality, intensity (quantified as μmol·m^−2^·s^−1^), and photoperiod duration. An imbalanced light distribution and mutual leaf shading can create a weak light environment, thus inhibiting photosynthesis. Long-term weak light conditions impair photosynthetic organ structure and reduce chlorophyll content [28]. In-plant lighting is an effective method to enhance photosynthetic efficiency, yield, and crop quality [29]. To balance production costs and resource efficiency, we implemented red–blue (7R2B) LED supplemental lighting in tomatoes, testing four spatial configurations: top lighting (T1), upward inter-canopy (T2), downward inter-canopy (T3), and bottom lighting (T4). We investigated the effects of these spatial configurations on the growth and development of tomato seedlings. Studies have shown that supplemental red–blue light promotes dry matter accumulation in tomatoes [30,31]. This is consistent with our research findings, which indicated a significant increase in biomass accumulation under different light spatial settings when compared to the CK treatment. In contrast, Trouwborst et al. reported that inter-plant supplementary lighting increased the dry matter distribution ratio in cucumber leaves while decreasing it in the fruit [32]. Paucek et al. confirmed that inter-plant lighting had no significant effect on the stem dry matter and leaf dry matter of tomatoes [33]. These contrasting results may be attributed to temperature differences between treatments caused by variations in supplementary lighting sources and methods. Compared to CK (without supplemental lighting), all spatial configurations of LED lighting significantly increased Chl *a* content in tomato leaves across different leaf layers, although the differences among treatments were not significant. The T1 and T2 treatments significantly increased Chl *b* and Total Chl content in leaf layer I, while the T3 and T4 treatments significantly increased Chl *b* and Total Chl content in leaf layers II and III, respectively. Leaves exposed to relatively high light intensity tend to have higher chlorophyll content. For instance, the upper leaves of the tomato canopy often exhibit greater chlorophyll content than the middle and lower leaves. Davies et al. indicate a positive correlation between leaf chlorophyll content and light absorption rate, with a more pronounced relationship in the green light spectrum [34]. This may explain the increased light absorption rate observed under inter-plant supplementary lighting treatment. In addition to chlorophyll content, we examined the precursors involved in chlorophyll synthesis. The results showed that, compared to the CK treatment, the T2 treatment significantly increased Proto IX content in leaf layer I, the T3 treatment significantly increased Pchlide content in leaf layer II, and the T4 treatment significantly increased precursor content in leaf layer III. These findings align with most previous studies, suggesting that the accumulation of chlorophyll content may initially depend on the synthesis of precursor substances [35]. This process enhances light energy absorption and transmission, improving overall photosynthetic efficiency in tomato plant foliage.

Pn is a primary indicator of photosynthetic capacity, which directly reflects a plant’s ability to photosynthesize [36]. The experimental results demonstrated that compared to the CK without supplemental lighting, the T1 treatment significantly increased Pn, Gs, and Tr in leaf layer I of tomato leaves. In contrast, the T4 treatment significantly enhanced Pn, Gs, and Tr in leaf layers II and III. These findings suggest that the T1 treatment improved gas exchange parameters in leaf layer I; however, due to shading effects within the tomato canopy, the T1 treatment had a less pronounced effect on gas exchange parameters in leaf layers II and III. Conversely, the T4 treatment, which involved illuminating the plants from below, enhanced gas exchange parameters in the lower leaf layers, although it resulted in lower gas exchange performance in leaf layer I compared to T1 treatment. Furthermore, all LED lighting spatial configurations significantly reduced Ci. Therefore, the T1 and T4 treatments enhanced photosynthetic capacity in tomato leaves and promoted CO_2_ fixation, consistent with findings in lettuce, tomato, and peony [13,37,38]. Chlorophyll fluorescence parameters ɸPSII and *qP*, as reliable indicators of photosystem II’s photochemical efficiency and reaction center openness, provide a comprehensive probe for photosynthesis, reflecting the absorption, conversion, transmission, and allocation of light energy by plants [39,40]. Under LED lighting, chlorophyll fluorescence parameters demonstrated positive responses. Plants capture light energy to generate chemical energy in the form of adenosine triphosphate (ATP) and nicotinamide adenine dinucleotide phosphate/reduced (NADPH), which drives the assimilation of nitrogen and the conversion of CO_2_ into sugars [41]. In studies assessing NPQ dynamics between top and bottom leaves, Tausz et al. reported faster NPQ induction in the bottom leaves, whereas Acevedo-Siaca et al. found no significant differences [42,43]. Yu et al. demonstrated that top lighting not only enhances the photosynthetic rate of leaves at various positions but also significantly increases ɸPSII and *qP* [44]. These increases suggest a greater demand for ATP and NADPH during the carbon assimilation process, aligning with the observed enhancement in net photosynthetic rate. In our study, the T1 treatment significantly increased the chlorophyll fluorescence parameters *qP*, rETR, *Fv/Fm*, and ɸPSII in leaf layer I and increased the NPQ in leaf layer III. The T4 treatment significantly enhanced *qP*, rETR, *Fv/Fm*, ɸPSII, and *Fv’/Fm’* in leaf layers II and III. The other treatments showed no significant effects on chlorophyll fluorescence parameters across the three layers, although all values were higher than those under the CK treatment (i.e., without supplemental lighting). Additionally, all LED lighting treatments significantly reduced the NPQ in leaf layers I, II, and III.

The Calvin cycle is central to carbon assimilation, playing a critical role in carbon fixation during photosynthesis [45,46]. This process primarily consists of light-independent redox reactions occurring in the chloroplast stroma, where absorbed CO_2_ is fixed. Rubisco catalyzes the carboxylation phase of the Calvin cycle, and the photosynthetic rate of plants is limited by the carboxylation activity of Rubisco and the regeneration capacity of ribulose-1,5-bisphosphate (RuBP) [47,48]. Chloroplastic isoforms of GAPDH catalyze the reduction of 1,3-bisphosphoglycerate, contributing to the Calvin cycle [49]. Enzymes such as FBPase, FBA, and TK are primarily involved in the RuBP regeneration process, converting glyceraldehyde-3-phosphate (G3P) and dihydroxyacetone phosphate (DHAP) into RuBP—the CO_2_ acceptor molecule [50]. The Calvin cycle, which facilitates CO_2_ fixation and energy storage during photosynthesis, is also influenced by LED light treatments. Studies have revealed that light of different quality can modulate photosynthesis by altering the activities of Calvin cycle enzymes in plant leaves [51]. In our study, all spatial LED light configurations increased the activities of key Calvin cycle enzymes—namely, Rubisco, FBA, GAPDH, FBPase, and TK—in tomato leaves across the upper, middle, and lower canopy layers. Specifically, the T1 treatment significantly enhanced the activity of Calvin cycle enzymes in the upper canopy leaves, while the T4 treatment had a more pronounced effect on FBA, FBPase, and TK activities in the lower canopy leaves, resulting in significant enzyme activity increases. This improvement facilitates CO_2_ fixation, accelerates the completion of energy storage in photosynthesis, and enhances the overall photosynthetic capacity of tomato plants. The results of this study are consistent with those of Qi et al., demonstrating that inter-plant supplemental lighting can enhance the photosynthetic capacity of middle and lower leaves to some extent [52]. However, compared to inter-plant lighting, top lighting has a more pronounced effect on improving overall plant photosynthetic capacity, which may be related to the accumulation of Rubisco activase (RCA) protein. RCA plays a crucial role in the activation and maintenance of Rubisco by facilitating the release of phosphorylated sugar inhibitors from its active sites, thereby enhancing Rubisco activity [53,54].

Previous studies have shown that supplementing red light with blue light can promote the production of photosynthetic products [30,55,56]. Specifically, LED red–blue (3R1B) light supplementation has been demonstrated to enhance SPS enzyme activity and sucrose accumulation in tomato leaves [15]. In our experiment, the various LED spatial light source configurations significantly increased the contents of photosynthetic products, such as glucose, fructose, and sucrose, particularly in leaf layers I and III. Among these, T4 significantly enhanced the levels of glucose, fructose, and sucrose in leaf layer III, which were markedly higher than those under the other treatments. Furthermore, compared to CK (without supplemental light), the LED spatial light source treatments significantly increased the activities of SS, SPS, AI, and NI enzymes in tomato leaves [57]. Bottom-up supplemental lighting notably enhanced the activities of sugar metabolism-related enzymes in the lower leaf layers, whereas top-down lighting prominently elevated these enzyme activities in the upper leaf layers. Comparative analysis of sugar metabolism pathways revealed that the LED spatial light source treatments enhanced SS, SPS, AI, and NI activities, thereby promoting the synthesis and accumulation of sucrose, fructose, and glucose. This improvement further boosted the photosynthetic capacity and carbohydrate content of tomato leaves [36]. Endogenous hormones play an indispensable role in the growth and development of plants [58,59]. Previous studies on crops such as tomatoes, cabbages, and grapes have indicated that LED supplementation significantly increases endogenous hormone levels [60,61]. Consistent with these findings, our research demonstrated that, compared to the control, all LED spatial light source configurations increased the IAA, ZT, and GA levels in tomato leaves while significantly reducing ABA levels. The top-down (T1) and bottom-up (T4) lighting treatments had the most pronounced effects on increasing IAA, ZT, and GA contents. This study highlights the significance of light management strategies in optimizing photosynthetic efficiency and provides theoretical support for enhancing supplemental lighting techniques in agricultural production. LED inter-plant supplemental lighting has been shown to increase leaf weight and photosynthetic capacity, particularly in middle and lower leaves, while also improving fruit yield, quality, and stress resistance [62]. Additionally, studies indicate that tomato upper leaves exhibit the highest light energy utilization efficiency and the strongest perception of light signals, followed by middle leaves [63]. However, a systematic comparison between inter-plant and top supplemental lighting remains limited, and the mechanisms by which supplemental lighting regulates photosynthesis and plant growth require further exploration. Given the lack of standardized LED supplemental lighting guidelines in tomato production, this study examines four LED lighting strategies targeting different plant positions in a controlled environment to evaluate their effects on leaf photosynthetic capacity, plant growth, and overall morphology. The findings aim to optimize LED supplemental lighting strategies to maximize both lighting efficiency and resource utilization, while future research should further investigate the effects of different lighting strategies on RCA regulation and Rubisco activity maintenance to deepen our understanding of the interactions between the light environment and photosynthetic enzyme activity.

## 4. Materials and Methods

### 4.1. Tomato Material and Growth Conditions

The experiment was conducted in a modern greenhouse located at Gansu Agricultural University. Tomato (*Solanum lycopersicum* L. cv. Jingfan 502) seeds were obtained from Jingyan Yinong Seed Sci-Tech Co., Ltd. (Beijing, China). Uniform and consistent seeds were selected and soaked in water at 28 °C for 8 h to ensure full hydration. Subsequently, the seeds were surface-sterilized in a 4% (*v*/*v*) sodium hypochlorite solution for 15 min and rinsed several times with sterile distilled water. Next, the seeds were evenly placed in culture dishes containing moist filter paper, covered with plastic wrap, and incubated in a dark climate chamber (RDN-400E-4; Ningbo Dongnan Instrument Co., Ltd., Ningbo, China) at 28 °C and 75% humidity for 30 h to induce germination. The germinated seeds were sown into 50-cell trays (54.0 cm long × 30.0 cm wide × 4.4 cm deep) filled with a mixture of peat, vermiculite, and perlite matrix (*v*/*v*/*v* = 2:1:1) and placed in an climate chamber—with a light cycle of 12 h/12 h (day/night), photon flux density of 320 μmol·m^−2^·s^−1^, temperature of 28 °C light/20 °C dark, and humidity of 30%—for seedling cultivation. When the seedlings had developed to the four-leaf stage, the uniform and robust seedlings were transplanted into the greenhouse and planted in plastic pots (20 cm × 20 cm × 26 cm; one plant per pot) and filled with 5.1 kg of identical substrate. Tomato plants were irrigated with full-strength Hoagland nutrient solution every 2 days.

### 4.2. Light Treatments and Experimental Design

**Figure 11:**
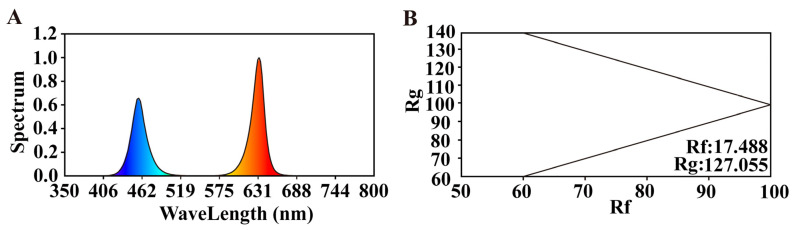
Spectral characteristics of the LED supplemental lighting system. (**A**) Spectral distribution of red (631 nm) and blue (462 nm) LEDs at a 7:2 ratio; (**B**) color quality metrics of the supplemental lighting for plant growth. Rg, Gamut Index; Rf, Fidelity Index.

This experiment utilized a strip LED light source (red/blue ratio = 7:2; HY-115CM-36×3W-RB; Shenzhen Houyi Energy Saving Technology Co., Ltd., Shenzhen, China), where the peak wavelengths of red and blue light were 640 nm and 450 nm, respectively. The light source, measuring 1.15 m in length, had a rated power of 108 W, light emission surfaces on both sides of the light source, and a maximum intensity of 19,130 lx. The LED spectrum is shown in Figure 11. Supplemental lighting treatments began in the sixth week after tomato transplantation. Tomato seedlings with uniform growth were selected and randomly divided into five groups, which were exposed to LED lighting for 30 days. The supplemental lighting was applied in the morning (6:00–9:00) and evening (17:00–20:00). The LED light tubes were placed in the middle of the plants, and the height of the light source was adjusted according to the growth of the tomato plants. To assess light distribution, light intensity at different canopy levels was measured using a light meter (PLA-30, EVERFINE Technology Co., Ltd., Hangzhou, China). The intensity was consistently maintained at 400 μmol·m^−2^·s^−1^ across all treatments. Measurements were conducted at multiple points within the canopy, including the upper, middle, and lower layers, to ensure an accurate representation of light distribution.

**Figure 12:**
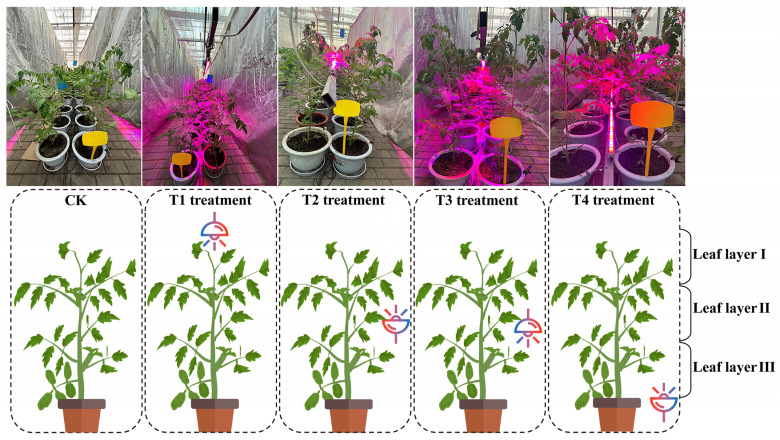
Schematic diagram of the spatial arrangement of LED light source installations for each treatment and the distribution of tomato leaf levels. The treatments include no supplementary lighting (CK), top-down lighting (T1), mid-canopy upward lighting (T2), mid-canopy downward lighting (T3), and bottom-up lighting (T4).

Five different installation heights were set in the experiment: the control group (CK), T1, T2, T3, and T4 treatments (see Figure 12). The CK treatment received no supplementary lighting. For the T1 treatment, the light source was placed above the top of the seedling canopy leaves of the tomato plants and kept at a distance of about 10 cm from the seedling canopy. In the T2 treatment, the light source was positioned at the same height as the first fruit cluster with an upward orientation, while the T3 treatment used a downward orientation at the same height. In the T4 treatment, the LED tube was positioned about 10 cm above the plant’s lowest leaves. Shading cloths were installed on both sides of each treatment to prevent light interference between treatments. The height of the light source was adjusted according to plant growth, ensuring a constant distance of 10 cm from the seedlings, with a maintained light intensity of 400 μmol·m^−2^s^−1^. After 30 days of illumination, 10 tomato plants were randomly selected from each treatment to measure relevant physiological indicators. The light distribution around the first cluster of fruits was considered the most representative, and the leaves of the plants were categorized into three layers for analysis: leaves located 4–6 nodes above the fruit, leaves between the upper and lower 2 nodes of the fruit, and leaves 4–6 nodes below the fruit. Leaves from each layer were randomly selected for measurement of growth and photosynthetic parameter measurements.

### 4.3. Measurement of Biomass and Specific Leaf Weight

After 30 days of treatment, six tomato plants were randomly selected from each group. The entire plants, including stems and roots, were harvested, inactivated at 105 °C for 30 min, and subsequently dried at 80 °C until a constant weight was achieved. The dry weights of the leaves, stems, and roots were determined using an electronic balance (precision: ±0.001 g; model: BCE224I-1CCN, Sartorius Co., Hamburg, Germany). For each treatment, three leaves from the same node position were sampled from the upper, middle, and lower leaf layers. These leaves were spread flat and scanned using a scanner (Win RHIZO Pro LA2400, Regent Instruments Inc., Quebec City, QC, Canada) to measure the corresponding leaf areas. The scanned leaves were then dried to a constant weight using the same procedure as described above. The SLW was calculated using the following formula.

SLW (mg cm^−2^) = Dry Weight/Leaf Area (1)

### 4.4. Determination of the Contents of Photosynthetic Pigments and Chlorophyll Synthesis Precursors

The Chl *a*, Chl *b*, Total Chl, and Car concentrations were determined using the acetone method [64]. Freshly shredded and mixed leaves (0.2 g) were selected from three leaf layers of tomato seedlings in different treatment groups and immersed within 10 mL of 80% (*v*/*v*) acetone reagent in darkness for 48 h (during which the test tubes were shaken periodically) until fully colorless leaves were obtained under room temperature. The optical density values of the extracted solutions were measured at 470, 645, and 663 nm using a spectrophotometer (UV-1780, Shimadzu Corp., Kyoto, Japan) and zeroed with acetone (80%). Each pigment’s content was calculated using the following equations, where V denotes the total volume of sample acetone extraction solution (mL) and FW denotes the sample’s fresh weight (FW, g):

Chl *a* (mg g^−1^ FW) = (12.21 × OD663 − 2.81 × OD645) × V/1000FW (2)

Chl *b* (mg g^−1^ FW) = (20.13 × OD645 − 5.03 × OD663) × V/1000FW (3)

Total Chl (mg g^−1^ FW) = Chl *a* + Chl *b*= (17.32 × OD645 + 7.18 × OD663) × V/1000FW (4)

Car (mg g^−1^ FW) = (4.7 × OD470 − 4.6764 × OD645 − 1.9386 × OD663) × V/1000FW (5)

Proto IX, Mg-Proto IX, and Pchlide were determined according to the method of Hodgins and Van Huystee, with slight modifications [65]. Fresh leaves were removed from the main veins and cut into pieces. A 0.15 g of the sample was taken and added to 10 mL of 80% alkaline acetone (acetone and 1% ammonia; *v*/*v* = 4:1). After grinding and extraction, the leaves were soaked in the dark until the tissue turned white. The extract was then centrifuged at 1500 g for 10 min. The absorbance of the supernatant was measured at wavelengths of 575 nm, 590 nm, and 628 nm, and the contents of the three intermediates were calculated using the corresponding formulae:

Proto IX (μmol g^−1^ FW) = (0.18016 × OD_575_ − 0.04036 × OD_628_ − 0.04515 × OD_590_) × V/FW (6)

Mg-Proto IX (μmol g^−1^ FW) = (0.06077 × OD_590_ − 0.01937 × OD_575_ − 0.003423 × OD_628_) × V/FW (7)

Pchlide (μmol g^−1^ FW) = (0.03563 × OD_628_ + 0.007225 × OD_590_ − 0.02955 × OD_575_) × V/FW (8)

### 4.5. Determination of Gas Exchange Parameters

Under stable light conditions between 9:00 and 11:00, gas exchange parameters of plants were measured using a portable photosynthesis system (CIRAS-2, PP Systems Inc., Amesbury, MA, USA). Measurements were taken from leaves at different canopy positions for each treatment by placing them in the leaf chamber. The parameters measured included Tr, Ci, Gs, and Pn. The instrument was set to the following conditions: relative humidity at 75%, ambient CO_2_ concentration of 380 μmol·mol^−1^, photosynthetic photon flux density (PPFD) from an integrated LED light source at 400 μmol·m^−2^·s^−1^, and leaf temperature at 25 °C.

### 4.6. Determination of Chlorophyll Fluorescence Parameters

The chlorophyll fluorescence parameters of fully expanded tomato leaves at different canopy positions under various treatments were measured using an Imaging-PAM chlorophyll fluorescence imaging system (Walz, Effeltrich, Germany). The measured parameters included the ETR, NPQ, *Fv*/*Fm*, *qP*, and ɸPSII. Prior to measurements, leaves were kept in the darkness for 30 min. The detection settings were as follows: measuring light intensity at 0.1 μmol·m^−2^·s^−1^, the intensity of the photochemical light was set to 111 μmol·m^−2^·s^−1^, saturation pulse intensity at 2700 μmol·m^−2^·s^−1^ with a duration of 0.8 s, and 20 s for the time interval. The initial fluorescence in the dark (*Fo*) and the maximum fluorescence yield in the dark (*Fm*) were obtained by applying a saturating pulse of light, allowing for the calculation of the *Fv*/*Fm*. After 300 s of actinic light exposure, the steady-state fluorescence yield (*Fs*) was recorded. Simultaneously, the maximum fluorescence yield under actinic light (*Fm*′) was measured using a saturating pulse of 0.8 s. When actinic light was turned off and far-red light was applied, the initial fluorescence yield under light (*Fo*′) was obtained. Related parameters were calculated using appropriate formulas [66].

*Fv*/*Fm* = (*Fm* − *Fo*)/*Fm* (9)

*Fv*′/*Fm*′= (*Fv*′ − *Fo*′) *Fm*′ (10)

ɸPSII = (*Fm*′ − *Fs*)/*Fm*′ (11)

*qP* = (*Fm*′ − *Fs*)/(*Fm*′ − *Fo*′) (12)

ETR= (*Fm*′ − *Fs*)/*Fm*′ × PAR × 0.5 × 0.84, where PAR = 300 μmol·m^−2^·s^−1^. (13)

### 4.7. Enzyme Activities Related to Carbon Metabolism in Tomato Leaves

For each treatment, 12 functional leaves of uniform size and located at the same node were collected from different canopy layers. The leaves were cut into small pieces and rapidly frozen in liquid nitrogen. Precisely 1 g of leaf tissue was homogenized with 9 mL of pre-cooled phosphate-buffered saline (PBS, pH 7.4) at 4 °C. The homogenate was then centrifuged at 4 °C and 5000 rpm for 25 min. The activities of key enzymes involved in carbon metabolism—including Rubisco, FBPase, TK, GAPDH, FBA, SS, SPS, AI, and NI—were measured using enzyme-linked immunosorbent assay (ELISA) kits (Yaji Biotechnology Co., Ltd., Shanghai, China), following the manufacturer’s instructions. Appropriate reaction reagents were added to the collected supernatant, and the absorbance at 450 nm was measured using a microplate reader (Spectra Max C Max Plus, Molecular Devices, San Jose, CA, USA). Enzyme activity concentrations were calculated based on standard curves and expressed in international units per liter (IU/L).

### 4.8. Carbohydrate Content of Tomato Leaves

The determination of photosynthetic products was conducted following the method described by Georgelis et al. [67], with minor modifications. The glucose, fructose, and sucrose contents in the leaf samples were analyzed using high-performance liquid chromatography (HPLC). The process involved grinding 5 g of fresh leaves and transferring the homogenate to a 25 mL volumetric flask, followed by dilution with ultrapure water to the calibration mark. The mixture was then filtered into a 50 mL centrifuge tube and centrifuged at 4 °C and 10,000 r/min for 10 min. Subsequently, 2 mL of the supernatant was further filtered using a 0.22 µm water filter. The resulting filtrate was used to determine the glucose, fructose, and sucrose concentrations. The analysis was performed on an HPLC system (Agilent 1100, Santa Clara, CA, USA) with a differential refractive index detector. Chromatographic separation was achieved using an LC-NH_2_ amino column (460 mm × 250 mm). The mobile phase consisted of acetonitrile and water in a volumetric ratio of 3:1, with a 1.0 mL/min flow rate. The column temperature was maintained at 30 °C, and the injection volume was 20 µL.

### 4.9. Determination of Hormone Contents

The determination of hormone contents in tomato leaves was carried out based on the method described by Dobrev and Vankova [68], with some modifications. Fresh samples (0.5 g) were accurately weighed and ground into powder in liquid nitrogen. Five milliliters of extraction solution (hydrochloric acid/n-propanol/distilled water = 0.002:2:1, *v*/*v*/*v*) was added to the powdered sample. The mixture was homogenized and shaken at 100 rpm for 30 min at 4 °C. Subsequently, 2 mL of dichloromethane was added, and the mixture was shaken for another 30 min under the same conditions. Following centrifugation at 13,000 rpm for 10 min at 4 °C, 2 mL of the lower phase was transferred to a 5 mL centrifuge tube, concentrated, and reconstituted with 200 μL of a 50% methanol–water solution. The solution was filtered through a 0.22 μm organic filter membrane and analyzed using HPLC (Agilent 1100, Agilent Technologies, Ltd., Waldbronn, Germany) equipped with a ZORBAX Eclipse XDB C18 column (4.6 mm × 280 mm; 5.0 μm). The mobile phase consisted of 0.1% phosphoric acid solution and methanol (1:9, *v*/*v*), with a flow rate of 1.0 mL/min and an injection volume of 10 μL.

### 4.10. The Formula for the Affiliation Function Method

Affiliation value = (X − X min)/(X max − X min)(14)

Value of the inverse affiliation function = 1 − (X − X min)/(X max − X min)(15)

where X is the measured value, X max is the maximum value, and X min is the minimum value. The values were summed and averaged [69].

### 4.11. Statistical Analysis

Data were collected from at least three independent experiments, with each replicate comprising 6–12 plants. Statistical analyses were conducted using one-way analysis of variance (ANOVA), followed by Duncan’s multiple range test to determine significant differences among groups, with statistical significance set at *p* < 0.05. Correlation analysis, principal component analysis (PCA), and hierarchical cluster analysis (HCA) were conducted using Origin 2024 (OriginLab Corporation, Northampton, MA, USA) and the SPSS software (version 27.0; IBM Corp., Armonk, NY, USA). Figures were generated using GraphPad Prism 9.5 and TBtools (version 2.210).

## 5. Conclusions

In facility-based tomato production, weakly lit environments require supplementary lighting (e.g., LED) to enhance plant growth. The spatial arrangement of different LED light sources significantly influences their effects on various plant parameters. Our study investigated the impacts of various LED spatial configurations on the photosynthetic efficiency of tomato leaves. The results showed that the different spatial arrangements of LED supplementary lighting significantly enhanced photosynthetic capacity and photosynthetic product accumulation and increased the specific leaf weight of irradiated tomato leaves, thus promoting dry matter accumulation. Specifically, the T1 treatment significantly increased the synthesis of chlorophyll precursors in leaf layers I and II, leading to higher chlorophyll content, improved gas exchange parameters, reduced intercellular CO_2_ concentration, and decreased NPQ. The T4 treatment significantly boosted chlorophyll content in leaf layer III, enhancing electron transport capacity and promoting carbon dioxide assimilation. This enhancement was associated with the activation of key Calvin cycle enzymes, including Rubisco, FBA, GAPDH, FBPase, and TK, thereby improving photosynthetic efficiency and sucrose storage. These findings highlight the crucial role of the spatial configuration of LEDs in optimizing light distribution and photosynthetic performance, providing valuable insights for improved tomato production in protected cultivation systems.

## Data Availability

Data are available from the corresponding author.

## Supplementary Materials

The following supporting information can be downloaded at https://www.mdpi.com/article/10.3390/plants14091369/s1: Figure S1: Cluster analysis of the physiological indicators in tomato for different light treatment; Table S1: Affiliation function evaluation and composite rankings of photosynthesis-related physiological indexes.
