# Supplementary material for: Different Spatial Configurations of LED Light Sources Enhance Growth in Tomato Seedlings by Influencing Photosynthesis, CO2 Assimilation, and Endogenous Hormones
Source: Plants (Basel). 2025 Apr 30;14(9):1369. doi: 10.3390/plants14091369 (PMC12073193; doi:10.3390/plants14091369)
Supplement: Supplementary file 1 [file plants-14-01369-s001.zip › plants-3546966-supplementary.pdf]

Supplementary information.

Figure. S1. Cluster analysis of the physiological indicators in tomato for different light treatment.

Table S1 Affiliation functions evaluation and composite rankings of Photosynthetic related physiological indexes.

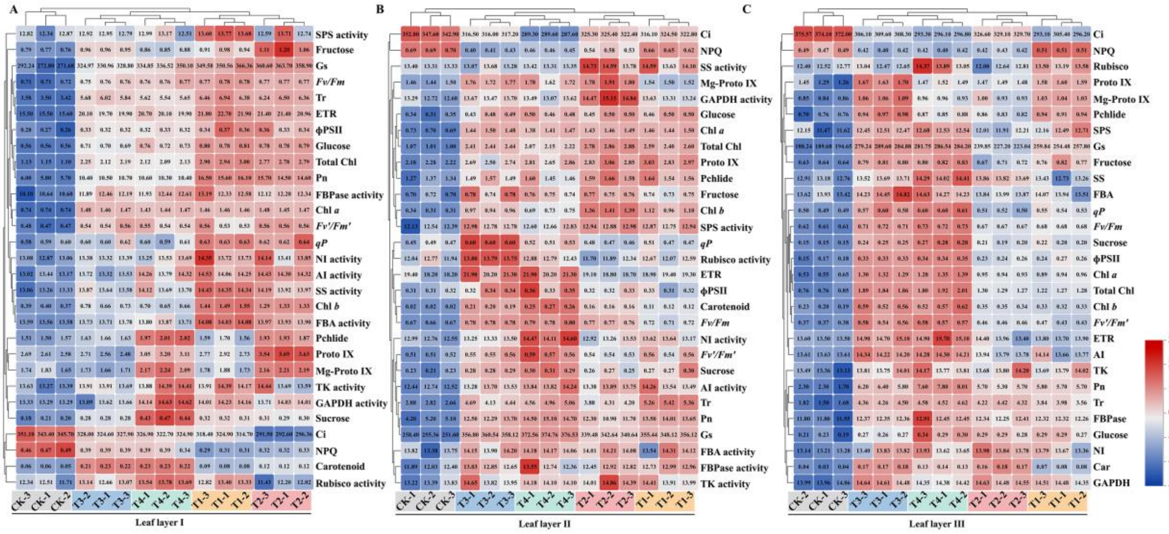

Figure S1. Cluster analysis of the physiological indicators in tomato for different light treatment. (A) Leaf layer I; (B) Leaf layer II; (C) Leaf layer III. Values are presented as mean ± SD (n = 3), derived from three independent biological replicates. For each biological replicate, at least six leaves were sampled. Statistical differences among treatments were evaluated using a significance threshold of $p < 0.05$. The physiological indicators include: Chl *a*; Chl *b*; Total Chl; Car; Proto IX; Mg-Proto IX; Pchlide; Pn; Tr; Ci; Gs; *Fv/Fm*; *Fv'/Fm'*; ϕPSII; NPQ; *qP*; ETR; Rubisco; GAPDH; FBA; FBPase; TK, transketolase; Glucose; Fructose; Sucrose; SPS; SS; AI; NI.

Table S1 Affiliation functions evaluation and composite rankings of Photosynthetic related physiological indexes.

**Value of the affiliation function**	**Treatment**	**CK**	**T1**	**T2**	**T3**	**T4**
Leaf layer I	X1	-1.2522	1.3371	0.6011	-0.1884	-0.4975
	X2	-1.2107	-0.5978	-0.1900	1.2673	0.7312
	X3	-0.3836	-0.6718	1.1581	-1.0650	0.9624
	μ1	0	1	0.7158	0.4109	0.2915
	μ2	0	0.2473	0.4119	1	0.7836
	μ3	0.3065	0.1768	1	0	0.9119
	D	0.0304	0.8020	0.6970	0.4611	0.4291
	Ranking	5	1	2	3	4
Leaf layer II	X1	-1.3941	0.8201	0.9820	0.20613	-0.6141
	X2	-1.0111	0.1856	-0.5287	-0.2574	1.61164
	X3	-0.4090	-0.3220	-0.8061	1.7427	-0.2056
	μ1	0	0.9319	1	0.6735	0.3283
	μ2	0	0.4563	0.1839	0.2874	1
	μ3	0.1558	0.1899	0	1	0.2356
	D	0.0127	0.7739	0.7512	0.6209	0.4585
	Ranking	5	1	2	3	4
Leaf layer III	X1	-0.7961	-0.1534	-1.0239	0.5698	1.4035
	X2	-1.3352	1.13608	0.2692	0.6175	-0.6876
	X3	-0.7919	-1.1363	1.38458	0.1799	0.3638
	μ1	0.0939	0.3586	0	0.6566	1
	μ2	0	1	0.6492	0.7902	0.2621
	μ3	0.1367	0	1	0.5221	0.5951
	D	0.0878	0.3949	0.1410	0.6596	0.8957
	Ranking	5	3	4	2	1

Note: Value of each comprehensive indicator (X_i), subordinate function values (μ_i), comprehensive evaluation value(D), and rank for tomato.
